# Hepatitis C Virus Dysregulates Polyamine and Proline Metabolism and Perturbs the Urea Cycle

**DOI:** 10.3390/cells13121036

**Published:** 2024-06-14

**Authors:** Natalia F. Zakirova, Olga A. Khomich, Olga A. Smirnova, Jennifer Molle, Sarah Duponchel, Dmitry V. Yanvarev, Vladimir T. Valuev-Elliston, Lea Monnier, Boyan Grigorov, Olga N. Ivanova, Inna L. Karpenko, Mikhail V. Golikov, Cedric Bovet, Barbara Rindlisbacher, Alex R. Khomutov, Sergey N. Kochetkov, Birke Bartosch, Alexander V. Ivanov

**Affiliations:** 1 Engelhardt Institute of Molecular Biology, Russian Academy of Sciences, 119991 Moscow, Russia; nat_zakirova@mail.ru (N.F.Z.); oakhomich@gmail.com (O.A.K.); o.smirnova.imb@gmail.com (O.A.S.); yanvarev@eimb.ru (D.V.Y.); gansfaust@mail.ru (V.T.V.-E.); olgaum@yandex.ru (O.N.I.); ilkzkil@gmail.com (I.L.K.); cool.mik3492594@yandex.ru (M.V.G.); alexkhom@list.ru (A.R.K.); snk1952@gmail.com (S.N.K.); 2 INSERM 1052, CNRS 5286, Centre Léon Bérard, Centre de Recherche en Cancérologie de Lyon, Université Claude Bernard Lyon 1, 69434 Lyon, France; jennifer.molle@inserm.fr (J.M.); lea_monnier@yahoo.com (L.M.); boyan.grigorov@inserm.fr (B.G.); birke.bartosch@inserm.fr (B.B.); 3 University Institute of Clinical Chemistry, Inselspital, Bern University Hospital, University of Bern, 3010 Bern, Switzerland; cedric.bovet@protonmail.com (C.B.); barbara.rindlisbacher@medics.ch (B.R.)

**Keywords:** hepatitis C virus, polyamines, urea cycle, proline metabolism, antiviral agents

## Abstract

Hepatitis C virus (HCV) is an oncogenic virus that causes chronic liver disease in more than 80% of patients. During the last decade, efficient direct-acting antivirals were introduced into clinical practice. However, clearance of the virus does not reduce the risk of end-stage liver diseases to the level observed in patients who have never been infected. So, investigation of HCV pathogenesis is still warranted. Virus-induced changes in cell metabolism contribute to the development of HCV-associated liver pathologies. Here, we studied the impact of the virus on the metabolism of polyamines and proline as well as on the urea cycle, which plays a crucial role in liver function. It was found that HCV strongly suppresses the expression of arginase, a key enzyme of the urea cycle, leading to the accumulation of arginine, and up-regulates proline oxidase with a concomitant decrease in proline concentrations. The addition of exogenous proline moderately suppressed viral replication. HCV up-regulated transcription but suppressed protein levels of polyamine-metabolizing enzymes. This resulted in a decrease in polyamine content in infected cells. Finally, compounds targeting polyamine metabolism demonstrated pronounced antiviral activity, pointing to spermine and spermidine as compounds affecting HCV replication. These data expand our understanding of HCV’s imprint on cell metabolism.

## 1. Introduction

Hepatitis C virus (HCV) is responsible for 9% of chronic liver disease cases worldwide [1]. Currently, the World Health Organization estimates 58 million people to be chronically infected with this virus [2]. Chronic hepatitis C (CHC) is often accompanied by persistent liver inflammation and the gradual development of fibrosis [3]. As a result, 10–30% of CHC patients will progress to liver cirrhosis within a 20–30-year period [2,3,4]. Patients with liver cirrhosis have substantial risks of hepatic decompensation and hepatocellular carcinoma. HCV infection is the cause of death of almost 400,000 patients with end-stage liver disease each year [1]. Combinations of direct-acting antivirals (DAAs) can clear the infection in almost every patient, though some CHC carriers with genotype 3 of the virus or with decompensated cirrhosis may not achieve sustained virological responses (SVR) [5,6]. While the high price of anti-HCV drugs still limits access to therapy in some parts of the world, insufficient screening allows between 68 and 94% of chronic hepatitis C cases to remain undiagnosed [7]. Moreover, SVR upon DAA treatment does not reduce the risk of liver cirrhosis and cancer to the level in the uninfected population [8]. Therefore, more insight into HCV pathogenesis is needed in order to develop drugs that may suppress or prevent liver dysfunction. One of the directions in this area is the investigation of virus-induced changes in the metabolism of infected cells.

HCV infection drives fibrogenesis and hepatocarcinogenesis by multiple mechanisms. These include chronic inflammation [9], production of profibrotic cytokines (i.e., TGFβ1) [10], interference of viral proteins with multiple signalling pathways in infected hepatocytes and concomitant inhibition of apoptosis and increased cell survival [11], overproduction of reactive oxygen species (ROS), dysregulation of antioxidant defence systems [12,13], and alteration of metabolic pathways [14,15]. HCV was reported to perturb multiple metabolic pathways, including glycolysis, glutaminolysis, and amino acid metabolism. Specifically, this virus increases rates of glycolysis and lactate production [16,17,18] and enhances consumption of glutamine and its conversion into α-ketoglutarate to drive [19] the tricarbonic acid (TCA) cycle in a tumour-like fashion [20]. HCV also increases levels of nucleos(t)ides, some amino acids, and fatty acids, and decreases intracellular concentration of AcCoA [16,18,21]. Replication of the virus also relies on metabolites such as sphingomyelin, which is critical for the formation of double-membrane vesicles where the virus replicase is localized [22]. However, the status of many other important metabolic pathways in HCV-infected hepatocytes remains unknown.

**Scheme 1:**
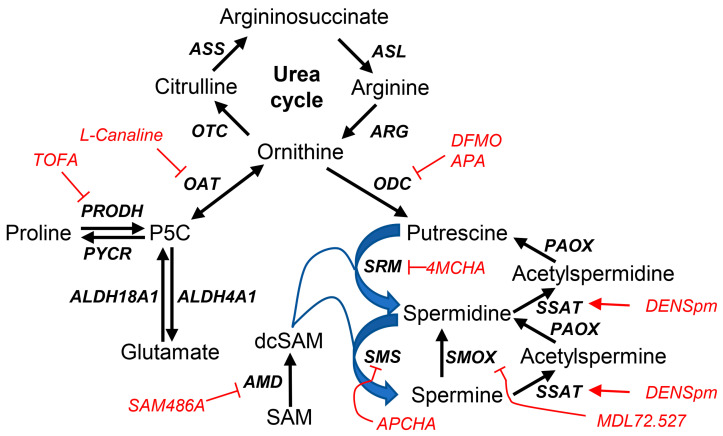
Scheme of the metabolism of biogenic polyamines and adjacent pathways and their pharmacological inhibitors used in this study. Compounds that target metabolic enzymes are presented in red. ARG—arginase, OTC—ornithine transcarbamoylase, ASS—argininosuccinate synthase, ASL—argininosuccinate lyase, ODC—ornithine decarboxylase, AMD—S-adenosylmethionine decarboxylase, SRM—spermidine synthase, SMS—spermine synthase, SSAT—spermidine/spermine-N^1^-acetyltransferase, PAOX—acetylpolyamine oxidase, SMOX—spermine oxidase, PRODH—proline dehydrogenase, P5C—Δ^1^-pyrrolidine-5-carboxylate, PYCR—P5C reductase, ALDH—aldehyde dehydrogenase.

Biogenic polyamines are low-molecular-weight compounds that carry several positively charged amino groups and thus can interact electrostatically with negatively charged nucleic acids and proteins. Spermine and spermidine are found in all types of eukaryotic cells at high concentrations, with their levels generally correlating with cell proliferation rates [23]. Polyamines are synthesized from ornithine, a metabolite of the urea cycle that is also linked to proline and glutamine/glutamate metabolic pathways (Scheme 1) [24,25]. The rate-limiting step of polyamine biosynthesis is the conversion of ornithine into diamine putrescine (1,4-diaminobutane), catalysed by ornithine decarboxylase (ODC). Putrescine is then converted into spermidine and spermine by spermidine and spermine synthases (SRM, SMS), respectively, with decarboxylated S-adenosyl methionine (dcSAM) being provided by S-adenosylmethionine decarboxylase (AMD). Polyamine degradation is achieved by two alternative pathways. The first one is a two-step process of acetylation by spermidine/spermine-N^1^-acetyl transferase (SSAT) with oxidation of acetylated spermine and spermidine by acetylpolyamine oxidase (PAOX) into spermidine and putrescine, respectively. An alternative pathway is a direct oxidation of spermine into spermidine by spermine oxidase (SMOX). Cells maintain levels of spermine and spermidine by multi-level control of expression of ODC, SSAT, SMOX, and, to some extent, AMD. Increased polyamine concentrations are a hallmark of hyperproliferative disorders such as cancer and autoimmune diseases [26]. At the same time, increased expression of SMOX that produces hydrogen peroxide and toxic acrolein is a feature of stroke and acute pancreatitis or is the key factor of carcinogenesis during *Helicobacter pylori* or *Bacteroides fragilis* infections [27,28]. Thus, targeting polyamine-metabolizing enzymes with pharmacologic inhibitors/activators is considered a promising strategy for the treatment of cancer. An example is the considerable progress that was achieved in the treatment of neuroblastoma by the addition of ODC inhibitor difluoromethylornithine (DFMO) to the standard combination of drugs [29,30].

However, much less is known about the interplay between polyamines and viral infections [24]. Previously, we reported that transient expression of HCV core and NS5A proteins affects the expression of the key polyamine-metabolizing enzymes via enhanced ROS production [31]. Moreover, a stable Huh7 cell line harbouring a full-length HCV replicon exhibited suppressed expression of ODC and SSAT, up-regulated expression of SMOX, and diminished levels of spermine and spermidine. However, all this was shown in a non-infectious system and warrants investigation in a model that ensures all steps of the viral life cycle. So far, nothing is known about whether polyamines are important for HCV replication, although other viruses have been shown to rely on spermine and spermidine at various stages of their life cycles [32]. The primary goal of this project was to investigate if the hepatitis C virus interferes with the metabolism of biogenic polyamines and related metabolites and if compounds that target polyamine-metabolizing enzymes affect HCV replication.

## 2. Materials and Methods

### 2.1. Reagents

Difluoromethylornithine (DFMO) was a kind gift from Prof. P. Woster (Medical University of South Carolina, Charleston, SC, USA). N,N′-bis(2,3-butadienyl)-1,4-butanediamine (MDL72.527), deferiprone, ciclopirox, and (S)-(+)-5-oxo tetrahydrofuran-2-carboxylic acid (THFA) were purchased from Sigma (St. Louis, MO, USA), and L-canaline from Caiman Chemical (Ann Arbor, MI, USA), while N^1^,N^11^-diethylnorspermine (DENSpm), N^1^-guanyl-1,7-diaminoheptane (GC7), N-(3-aminopropyl)cyclohexylamine (APCHA), and trans-4-methyl-cyclohecylamine (4MCHA) were supplied by Santa-Cruz Biotechnologies (Dallas, TX, USA). Sardomoside (SAM486A) was purchased from Cayman Chemicals (Ann Arbor, MI, USA). N^1^-Ethyl-N^11^-(cyclopropyl)-methyl-4,8-diazaundecane (CPENSpm) and N^1^-ethyl-N^11^-(cycloheptyl)-methyl-4,8-diazaundecane (CHENSpm) were kindly given by Dr Tuomo Keinanen (University of Eastern Finland, Kuopio, Finland). 3-Aminooxy-1-aminopropane (APA) was synthesized as described in [33]. DMEM and DMEM-F12 media were from Life Technologies (Carlsbad, CA, USA). Plasmax medium was assembled as described earlier [34,35]. [^14^C]-labelled ornithine and AcCoA were from GE Healthcare (Little Chalfont, UK). All other reagents were provided by Sigma. Oligonucleotides were synthesized by Evrogen J.S.C. (Moscow, Russia).

Huh7.5 cells and the plasmids pJFH1 and pSGR-JFH1 were kindly provided by Prof. C.M. Rice (The Rockefeller University, New York, NY, USA), Prof. Takaji Wakita (National Institute of Infectious Diseases, Tokyo, Japan), and Apath L.L.C. (Brooklyn, NY, USA).

### 2.2. Cells and HCV Infection Systems

Huh7.5 cells were cultivated in a high-glucose Dulbecco’s modified Eagle’s medium (DMEM) with GlutaMax supplemented with 10% foetal bovine serum (Biosera, Cholet, France) in a humid atmosphere at 37 °C and split every three days. Alternatively, the cells were maintained in a physiological Plasmax medium, as described previously [34].

#### 2.2.1. HCV Cell Culture (HCVcc) System

HCV viral stock was prepared using a plasmid pJFH1 according to a standard protocol [36]. Huh7.5 cells were seeded in DMEM-F12 medium on 6- or 12-well plates or 6 cm dishes 24 h before infection at a density of 4 × 10^5^ cells/well of a 6-well plate. When the cells reached 70% confluency, they were infected at 0.1 MOI. Four hours post-infection, the medium was changed to a fresh one, and the cells were kept for 3–10 days (as specified in the figure legends). The cells were harvested by scraping. During analysis of antiviral activity, compounds were added 4 h before infection and 4 h post-infection (upon change of virion-containing medium).

#### 2.2.2. HCV Replicon

The subgenomic HCV replicon corresponding to the JFH1 isolate was obtained by synthesis of the viral RNA via in vitro transcription from the plasmid pSGR-JFH1, and delivering it to Huh7.5 cells by electroporation, as described in [37]. The cells were maintained in DMEM-F12 supplemented with G418 (500 ng/mL) for ten passages prior to analysis. Later, when required, the cells were maintained in a Plasmax medium for at least three passages prior to analysis.

To assess the role of polyamines in HCV replication, the cells harbouring the replicon were seeded in DMEM with standard dialyzed serum (Gemini Bio, West Sacramento, CA, USA) supplemented with 1 mM aminoguanidine and, if required, a mixture of spermine and spermidine (100 µM each).

### 2.3. Quantification of Metabolites

#### 2.3.1. Polyamines

The cells were grown on 6-well plates. After removal of the culture medium, cells were washed twice with 1 mL PBS at 0 °C. Then, 0.1 mL mQ was added per well, and the plate was subjected to three cycles of freezing (−196 °C) and thawing at +4 °C. After the final thaw, samples were transferred to 0.5 mL polyethylene tubes. Each well was washed up with an additional 0.1 mL mQ, combined with the crude cell lysates and subjected to ultrasound treatment for 3 min at 0–4 °C. Debris was removed by centrifugation for 10 min at 14,000× *g*. Total protein content in supernatants was measured using the Pierce™ BCA Protein Assay Kit (Thermo Scientific, Waltham, MA, USA) prior to precipitation of protein by the addition of 60% perchloric acid to the final 3% concentration, followed by clarification by centrifugation for 10 min at 14,000× *g*. The clarified lysates were lyophilised and kept at −80 °C prior to analysis.

Polyamines were quantified by high-pressure liquid chromatography (HPLC) with precolumn derivatization with dansyl chloride. The analysis was performed by dissolving dry samples in 100 µL of water saturated at 20 °C Na_2_CO_3_. A different ratio mixture of 1,6-diaminohexane and 1,7-diaminoheptane was used as an internal standard, and hydrazine hydrate (2 µL) was applied to quench the dansylation reaction.

The solution of the dansylated polyamines in toluene (after 2-step extraction with 200 µL of toluene) was vacuum dried, and the residue was dissolved in 200 µL of methanol and applied on a reversed-phase column (Cosmosil C18-MS-II, 250 × 4.6 mm, 5 µm, 100 Å). The column was eluted (1 mL/min) with the following gradient: 0 min—0% B; 5 min—0% B; 60 min—100% B; 65 min—100% B, 70 min—0% B, 75 min—0% B. System A—30% acetonitrile, 69.5% H_2_O, 0.5% propionic acid. System B—79.5% acetonitrile, 20% tetrahydrofuran, 0.5% propionic acid. Column temperature 40 °C, pressure 80–120 bar, fluorescent detection: λ_ex_ 340 nm, λ_em_ 530 nm (detector RF-20A, Shimadzu Scientific Instrument, Columbia, MD, USA).

#### 2.3.2. Arginine and Proline

Arginine and proline levels of the cell extracts were measured by a nontargeted metabolic profiling approach based on high-resolution mass spectrometry (HRMS), as previously described [38]. Prior to HRMS analysis, the cell extracts were vacuum dried using a Savant SpeedVac Concentrator SPD101 (Thermo, Geneva, Switzerland) and resuspended in 200 µL 1:1 acetonitrile–methanol (*v*/*v*) and transferred to total recovery LCMS vials. Data processing and statistical analysis were performed as in [38]. Metabolic features were annotated based on their mass accuracy, isotopic pattern, and MS/MS fragments, if available. Amino acid levels in infected cells were normalized to levels in uninfected cells cultivated for the same time.

### 2.4. Enzyme Activity Assays

Enzymatic activity of spermidine/spermine-N^1^-acetyltransferase was determined according to the classical procedure of P.R. Libby [39], while ornithine decarboxylase activity was measured as described by J. Janne et al. [40].

### 2.5. Western Blotting

The cells were grown on 6-well plates or 6 cm dishes, harvested by scraping, lysed in RIPA buffer, and protein concentrations in lysates were measured using a BCA kit (Thermo Fischer Scientific, Waltham, MA, USA). Ten micrograms of total protein were applied on 10–12% sodium dodecyl sulphate-polyacrylamide gel, and immunoblotting was further carried out as described previously [41]. Primary murine antibodies to β-actin (ab3280, 1:500), ASS (ab124465, 1:2000), and ODC1 (ab193338, 1:1000), as well as rabbit antibodies to ASL1 (ab97370, 1:2000), were from Abcam (Cambridge, UK); rabbit antibodies to arginase I (9819s, 1:1000) and SSAT (61586, 1:200) were obtained from Cell Signaling Technology (Danvers, MA, USA), and antibodies to SMOX (hpa047117, 1:1000) from Sigma (St. Louis, MO, USA). Murine antibodies to OAT (sc-374243, 1:500), PRODH (sc-376401, 1:200), and PYCR (sc-243722, 1:200) were from Santa Cruz Biotechnology, Inc. (Dallas, TX, USA). Secondary HRP-conjugated goat antibodies to mouse IgG (sc-2005, 1:3000) and goat antibodies to rabbit IgG (sc-2004, 1:3000) were from Santa Cruz Biotechnology. All antibodies were used in 1% non-fat dry milk (BioRad, Hercules, CA, USA) in PBS with 0.05% Tween 20 (PBS-T).

### 2.6. Immunofluorescence

Huh7.5 cells were seeded on 24-well plates, infected as described above, fixed with methanol–acetone mixture, and stained with rabbit sera raised to HCV NS3 and secondary FITC-labelled antibodies to rabbit immunoglobulins (F0382, Sigma). The cells were visualized by confocal microscopy as reported previously in [20] or using a Zoe fluorescent cell imaging system (Bio-Rad, Hercules, CA, USA).

### 2.7. Reverse Transcription and Real-Time PCR (RT-qPCR)

Real-time and quantitative PCR were carried out as described in [37] with minor modifications. Briefly, total RNA was purified using Extract RNA Reagent (Evrogen J.S.C., Moscow, Russia) and treated with a recombinant RNAse-free DNAse (Roche, Basel, Switzerland) using specifications from the manufacturers. cDNA synthesis was carried out with RevertAid reverse transcriptase (Thermo Scientific, Rockford, IL, USA) and a random hexamer primer according to standard protocols. PCR was performed using primers listed in Table S1 in a LightCycler 96 System (Roche, Basel, Switzerland). A standard reaction mixture (10 μL) contained cDNA equivalent to 10 ng total RNA, 0.8 μM of the respective primers, and qPCRmix-HS SYBR (Evrogen). The amplification conditions were 55 °C for 5 min, 95 °C for 10 min, followed by 40 cycles each at 95 °C for 10 s and 57 °C for 1 min (signal collection temperature). β-Glucoronidase (GUS) was used as a housekeeping gene. The results were analysed by the ∆∆Ct approach [42]. Briefly, RNA levels were first normalized to levels of GUS mRNA in each sample. These standardized values were then used to compare fold changes between samples of interest.

### 2.8. Statistical Analysis

All data are presented as means ± standard error of means (SEM). Statistical significance was determined by paired two-tailed *t*-test, analysis of variance (ANOVA) with Tukey post hoc test, or Dunnett’s test using Graphpad Prism (7.0). A *p*-value < 0.05 was considered statistically significant if not stated otherwise.

## 3. Results

### 3.1. Hepatitis C Virus Perturbs Metabolism of Biogenic Polyamines

**Figure 1:**
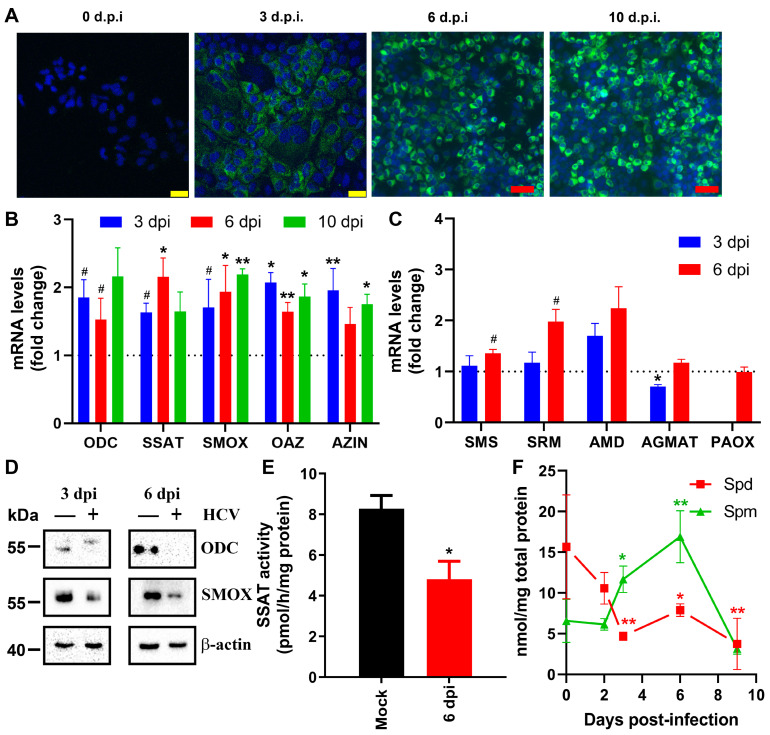
HCV infection suppresses the expression of polyamine-metabolizing enzymes at the posttranscriptional level. (**A**–**F**) Huh7.5 cells were infected with HCV at MOI 0.1. (**A**) Infection spread was monitored by immunofluorescence staining using anti-NS3 rabbit sera and FITC-labelled antirabbit antibodies; the nuclei were counterstained with DAPI, and signals were visualized by confocal microscopy at 63× magnification. The yellow scale bar corresponds to 25 µm; the red bars correspond to 50 µm. (**B**–**D**) Expression of polyamine-metabolizing proteins was assessed 3–10 days post-infection (dpi) by RT-qPCR (**B**,**C**) or Western blotting (**D**). Intracellular SSAT activity was quantified by monitoring the transfer of the ^14^C-labelled acetyl group from [^14^C]-AcCoA to the spermine molecule. (**F**) Polyamine levels were measured by HPLC. Graphs represent means ± SEM of four (**B**) or three (**C**,**E**,**F**) independent experiments. * *p* < 0.05, ** *p* < 0.01, ^#^ *p* < 0.1 compared to mock-infected cells by ANOVA with Dunnett’s post hoc test (**B**,**C**,**F**) or paired *t*-test (**E**).

Previously, we showed that transient overexpression of several HCV proteins increased the expression of the key polyamine-metabolizing enzymes [31]. However, long-lasting expression of HCV proteins from autonomously replicating viral RNA in human hepatoma Huh7 cells (i.e., replicon system [43]) decreased expression of ODC and SSAT and reduced polyamine content. In the present study, we aimed to unveil the imprint of the virus on polyamine metabolism in the infectious cell culture model (HCVcc). Huh7.5 cells were infected with HCV at a multiplicity of infection (MOI) of 0.1, and mRNA levels of most polyamine-metabolizing enzymes and regulatory proteins were assessed 3–10 days post-infection. The results shown in Figure 1B,C clearly show that HCV moderately up-regulates transcription of both biosynthetic and catabolic enzymes, although, for some of them, the analysis revealed only a trend towards statistical significance. As ornithine decarboxylase and spermidine/spermine-N^1^-acetyltransferase are regulated post-transcriptionally [44,45], levels of polyamine-metabolizing enzymes were also analysed by Western blotting (ODC, SMOX) or by measuring intracellular activity in cell lysates (SSAT). HCV infection induced a decrease in levels of these enzymes despite activation at the transcriptional level (Figure 1D,E). Quantification of polyamines revealed that HCV induces a time-dependent decrease in spermidine levels but an increase in spermine concentrations in a period of up to 6 days (Figure 1F). This correlates with down-regulation of polyamine biosynthesis and spermine catabolism. Levels of putrescine were below the detection limit (0.05 nmol/mg total protein). However, over longer infection periods, spermine levels were also decreased (Figure 1F), likely due to exhaustion of their total levels. These data clearly demonstrate that HCV infection triggers a shift of the spermine-to-spermidine ratio during the spread of the infection. However, at late stages of infection, spermine accumulation normalized to a level slightly below that observed in uninfected cells.

### 3.2. Hepatitis C Virus Down-Regulates Expression of Arginase I

**Figure 2:**
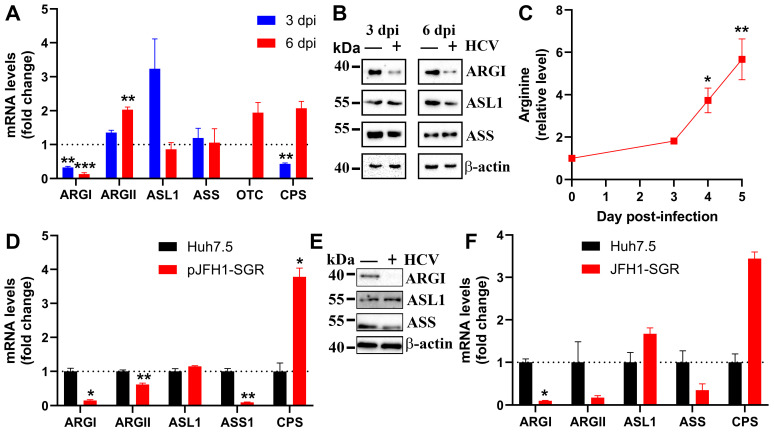
HCV interferes with the urea cycle by inhibiting the expression of arginase 1. (**A**–**C**) Huh7.5 cells were infected with an HCV inoculum at MOI 0.1 in DMEM-F12 media and harvested 3–6 days post-infection (d.p.i). (**D**–**F**) Alternatively, Huh7.5 cells stably transfected with subgenomic HCV RNA (replicon as equivalent for a chronic infection) or naïve Huh7.5 cells were maintained in DMEM-F12 (**D**,**E**) or Plasmax (**F**) media. Expression of urea cycle enzymes was assessed by RT-qPCR (**A**,**D**,**F**) or Western blotting (**B**,**E**). The β-actin subpanels from (**B**) are the same as in Figure 1D, as they are from the same experiment. (**C**) Intracellular arginine levels were quantified by LC-MS. The depicted values are relative, standardized to the levels measured in mock-infected cells. Graphs represent means ± SEM of three independent experiments, with the exception of (**A**), which shows the results of four independent experiments, and (**F**), which shows cells cultured for three passages in Plasmax medium (two experiments). * *p* < 0.05, ** *p* < 0.01, *** *p* < 0.001 compared to mock-infected cells or naïve Huh7.5 cells by ANOVA with Dunnett’s post hoc test (**A**,**C**) or paired *t*-test (**D**,**F**). Note that actin blots on Panel (**B**) were from the same experiment, as shown in Figure 1D.

Since polyamines are synthesized from ornithine, a non-proteinogenic amino acid of the urea cycle [24], our next step was to study the possible effects of the virus on the expression of urea cycle enzymes. Expression of arginase (ARG) I and II, ornithine transcarbamylase 1 (OTC), argininosuccinate synthase (ASS), and argininosuccinate lyase 1 (ASL1), as well as of carbamoylphosphate synthase (CPS1), was evaluated by RT-qPCR and/or Western blotting. This revealed that HCV down-regulates transcription of arginase 1 by several folds. Levels of extrahepatic arginase (ARGII) mRNA were increased at 6 dpi (Figure 2A), but ARGII was undetectable at the protein level by Western blotting. Expression of other genes did not show any significant changes. Transcripts of OTC in this cell line were either very low or even undetectable in some samples (Figure 2A). Next, we assessed levels of arginine. Mass spectrometry analysis indicated that HCV infection led to a time-dependent increase in the arginine pool in cells with suppressed ARGI expression.

**Figure 3:**
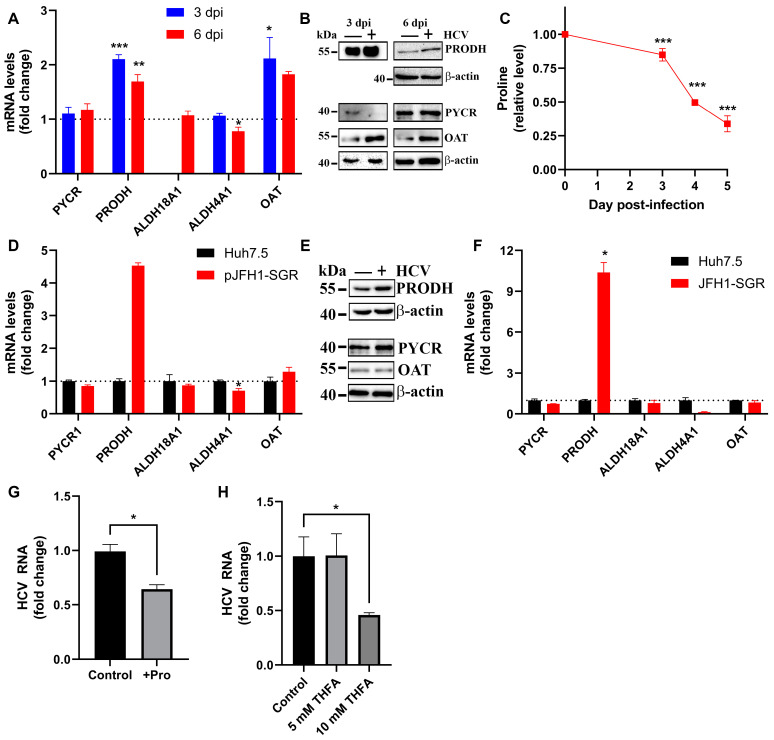
HCV induces proline catabolism by up-regulating proline dehydrogenase. (**A**–**C**) Huh7.5 cells were infected with HCV at MOI 0.1 in DMEM-F12 media. (**D**–**G**) Alternatively, Huh7.5 cells harbouring subgenomic HCV replicon or naïve Huh7.5 cells were maintained in DMEM-F12 (**D**,**E**) or Plasmax (**F**) media or DMEM-F12 supplemented with 2.5 mM proline (**G**). Expression of proline- pyrrolidine-5-carboxylate-metabolizing enzymes was assessed by RT-qPCR (**A**,**D**,**F**) or Western blotting (**B**,**E**). (**C**) Intracellular levels of proline were quantified by LC-MS, and values were normalized relative to the levels measured in mock-infected cells. (**H**) Huh7.5 cells were treated with THFA 4 h prior to infection and 4 h post-infection, with quantification of HCV RNA levels at 6 dpi. Graphs represent means ± SEM of three independent experiments, with the exception of (**F**) cells in Plasmax medium (two experiments). * *p* < 0.05, ** *p* < 0.01, *** *p* < 0.001 compared to mock-infected cells or naïve Huh7.5 cells by ANOVA with Dunnett’s post hoc test (**A**) or paired *t*-test (**D**,**F**). The subpanel with β-actin on panel (**E**) is the same as in Figure 2E, and subpanels from Figure 2B (3 dpi) are the same as in Figure 1D (3 dpi), as they are from the same experiments. The PRODH subpanel from (**B**) (3 dpi) corresponds to the bottom β-actin subpanel, while PRODH from 6 dpi infection has a separate β-actin blot.

To address the question of whether alteration of the urea cycle is a transient feature in HCV infection and occurs only shortly after virus entry, we assessed changes in the expression of urea cycle enzymes in cells harbouring subgenomic HCV replicons. Indeed, HCV also decreased ARGI levels in this model of chronic infection (Figure 3D,E). In this model, we also showed decreased expression of ASS and increased accumulation of ASL1. However, these latter changes were not observed in the HCVcc system.

Recently, Tardito’s group reported that the classical DMEM-F12 medium, which is often used in HCV research, has a profound imprint on cell metabolism [35]. Furthermore, triple-negative breast cancer cell lines in DMEM-F12 medium exhibit a reversed urea cycle, as arginine, present at non-physiologically high concentrations in DMEM-F12, is converted not into ornithine via arginase but into argininosuccinate via ASL1. Therefore, we verified our data using a physiological plasma-resembling Plasmax medium that has normal levels of arginine. Indeed, a subgenomic HCV replicon was found to suppress the expression of arginase I independently of the culture medium used (Figure 2D,F).

### 3.3. Hepatitis C Virus Up-Regulates Expression of Proline Dehydrogenase and Decreases the Intracellular Level of Proline

Next, we evaluated the effect of HCV on the metabolism of proline, as it can be converted into ornithine via the Δ^1^-pyrrolidine-5-carboxylate (P5C) intermediate (Scheme 1). These reactions are catalysed by proline dehydrogenase (PRODH, proline oxidase) and ornithine aminotransferase (OAT). Back-conversion of P5C into proline can be achieved by pyrroline-5-carboxylate reductase (PYCR). The initial screening of transcription rates for all these genes identified proline dehydrogenase (PRODH, proline oxidase) as a gene whose expression is up-regulated by the virus (Figure 3A). The levels of PRODH were elevated at the protein level as well (Figure 3B). The expression of PYCR that catalyses the reverse reaction was generally unaltered with the exception of decreased protein level at 3 days post-infection (Figure 3A,B). We also observed a moderate induction of OAT by the virus compared to mock-infected cells (Figure 3A,B). The induction of the proline-catabolizing enzyme resulted in a decrease in intracellular proline concentration (Figure 3C). An even more pronounced induction of PRODH was observed in Huh7.5 cells harbouring the subgenomic virus replicon, showing that the changes in proline catabolism are induced by the nonstructural viral proteins and suggesting that these changes also happen during long-lasting HCV infection (Figure 3D,E). Finally, as for the urea cycle, these changes occurred in both DMEM-F12 (Figure 3D) and Plasmax media (Figure 3F).

Since PRODH induction led to a decrease in intracellular proline concentration, our next step was to assess the impact of these changes on the replication of the virus. Proline levels in culture media are usually within the 0.15–0.20 mM range [46]. We therefore elevated its concentration approximately ten-fold, i.e., to 2.5 mM. However, this led only to a moderate (ap. 30%) reduction in replication levels in the subgenomic replicon model (Figure 3G), indicating that reduction of the proline pool in infected cells is dispensable for the virus. Similarly, a reduction in HCV replication levels was also achieved by treatment with the PRODH inhibitor (S)-(+)-5-oxotetrahydrofuran-2-carboxylic acid (THFA) (Figure 3H).

### 3.4. HCV Does Not Affect the Expression of the Genes That Channel Glutamate into Proline/Ornithine

Δ^1^-Pyrrolidine-5-carboxylate can be synthesized not only from proline but also from glutamate [47]. So, our next step was to assess the levels of ALDH18A1, which encodes Δ^1^-pyrrolidine-5-carboxylate synthase, an enzyme converting Glu into P5C, and ALDH4A1, which mediates the reverse reaction. However, RT-qPCR analysis showed no pronounced and statistically significant changes in the transcription of their genes in response to HCV infection in HCVcc (Figure 3A) and subgenomic replicon (Figure 3D,F) systems.

### 3.5. Inhibition of Polyamine Biosynthesis or Catabolism Suppresses HCV Replication

Our final step was to assess the importance of biogenic polyamines for HCV replication. It was performed by evaluating compounds that either inhibit or induce the key enzyme of polyamine metabolism as potential anti-HCV agents. These compounds included the inhibitors of ornithine decarboxylase (difluoromethylornithine, DFMO; 3-aminooxy-1-aminopropane, APA), polyamine oxidases (MDL72.527) or AMD (SAM486A), potent SSAT inducers N^1^,N^11^-diethylnorspermine (DENSpm) and N^1^-ethyl-N^11^-cyclopropylnorspermine (CPENS), inhibitors of eIF5a hypusination (a spermidine-dependent post-translational modification) GC7, deferiprone, and ciclopirox. In addition, we evaluated N-(3-aminopropyl)cyclohexylamine (APCHA) and trans-4-methyl-cyclohecylamine (4MCHA)—the inhibitors of spermine and spermidine synthases. As an additional control, we analysed another bis-alkylated spermine, N^1^-ethyl-N^11^-cycloheptylnorspermine (CHENS), which is a much weaker SSAT inducer [48]. Finally, we also tested L-canaline, which inhibits ornithine aminotransferase, the enzyme that catalyses the conversion of P5C into ornithine. It is noteworthy that the compounds were tested in non-toxic concentrations (Figure S1).

**Figure 4:**
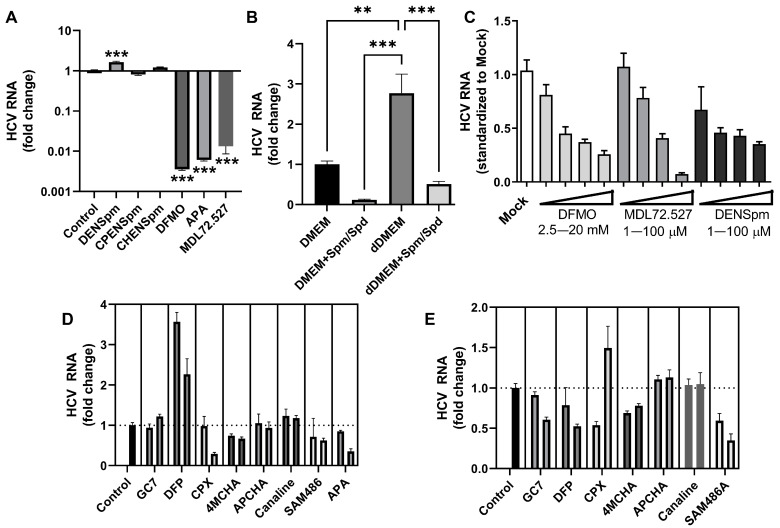
Inhibitors of ornithine decarboxylase and polyamine oxidases inhibit HCV replication. (**A**) Huh7.5 cells harbouring a subgenomic HCV replicon were treated with 20 µM DENSpm, CPENSpm, CHENSpm, or MDL72.527 for 3 days. (**B**) Huh7.5 subgenomic replicon-harbouring cells were cultivated in DMEM with standard (DMEM) or dialysed (dDMEM) FBS supplemented with 1 mM aminoguanidine alone or with a mixture of spermine and spermidine (100 µM each). (**C**,**D**) HCV-infected Huh7.5 cells were treated with DFMO, MDL72.527, DENSpm, or with GC7 (10 and 30 µM), deferiprone (DFP, 200 and 600 µM), ciclopirox (25 and 75 µM), trans-4-methyl-cyclohecylamine (4MCHA), N-(3-aminopropyl)cyclohexylamine (APCHA) or L-canaline (30 and 100 µM), or SAM486A (1 and 3 µM) for 6 days. (**E**) The subgenomic HCV replicon cell line was treated similarly for 3 days. HCV RNA levels were quantified by RT-qPCR. Graphs represent means ± SEM of three independent experiments. ** *p* < 0.01 and *** *p* < 0.001 by ANOVA with Dunnett’s post hoc test (**A**) or Tukey’s test (**B**).

Initially, these compounds were tested in Huh7.5 cells harbouring the full-length HCV replicon (Figure 4A,E) with further evaluation in the HCVcc system (Figure 4C,D). Quantification of RNA levels revealed that the inducers of SSAT (Figure 4A), inhibitors of polyamine synthases, and ornithine aminotransferase (Figure 4D,E) did not affect HCV replication. The hypusination pathway also seemed to be dispensable for HCV replication, as its classical selective inhibitor GC7 did not alter HCV RNA levels in either system, and other, less selective, inhibitors had the opposite impact in HCVcc and replicon models (Figure 4D,E).

At the same time, significant anti-HCV activity was displayed by DFMO, APA, and SAM486A, as well as MDL72.527, drugs that target polyamine biosynthesis and catabolism, respectively (Figure 4A,C,E). To analyse their mode of antiviral action, polyamine levels were quantified in treated naïve cells and the cells with HCV replicon. As can be seen from Table 1, treatment of naïve Huh7.5 cells with 20 µM DENSpm resulted in a 6.5-fold up-regulation of SSAT transcription and a concomitant 2.8-fold decrease in spermine levels. Similar changes were observed in the replicon-harbouring cells. The increase in SSAT expression was more pronounced in replicon-harbouring cells compared to naïve cells due to lower gene expression in the untreated cells, but the total activity level became comparable to the level in the treated naïve cells. Treatment of the naïve Huh7.5 cells with 1 mM DFMO suppressed ODC activity by just two-fold, with no changes in the levels of spermine or spermidine. Unfortunately, putrescine content in the hepatic cells was close to the limit of detection, and quantification was not possible in all samples. Strikingly, in the cells with the HCV replicon, DFMO increased the content of spermine and spermidine by 5–7-fold. Similar changes were seen for MDL72.527, which also exhibited antiviral activity. Since reduced HCV replication was observed in the samples with elevated polyamine content, the next step was to verify the influence of spermine and spermidine on HCV replication using other approaches. HCV RNA levels were evaluated in replicon-harbouring cells maintained either in a standard DMEM supplemented with both polyamines in combination with aminoguanidine, an inhibitor or their degradation by serum amino oxidases, or in a medium with a dialyzed serum (dDMEM), i.e., depleted of polar metabolites including polyamines. Indeed, in the presence of dialyzed serum, HCV replication was elevated, whereas exogenous polyamines suppressed it (Figure 4B). Thus, high polyamine content in infected cells suppresses viral replication, and HCV reduces spermine and spermidine pools to alleviate their negative effect.

## 4. Discussion

Polyamines are ubiquitous compounds critical for cell growth and differentiation. Changes in their metabolism are associated with the development of proliferative and metabolic disorders such as tumorigenesis and autoimmune pathologies (i.e., psoriasis) [49,50]. However, the significance of spermine and spermidine for viral infections was studied less extensively until recently. Several viruses, including Kaposi’s sarcoma-associated virus (KSAV) [51], Epstein–Barr virus (EBV) [52], and Dengue virus [53], have been shown to suppress the expression of SSAT at transcriptional or translational levels. KSAV also induces expression of ODC, although only at latent stages of replication in 2D culture [54] or in 3D models [51]. A similar, although transient, increase in ODC was described in 3D organoids infected with KSAV [51]. As a result, at least some of these infections increase polyamine content in infected cells. In contrast, SARS-CoV-2 [55], porcine reproductive and respiratory syndrome virus (PRRSV) [56], and porcine endemic diarrhoea virus (PEDV) [57] up-regulate expression of SSAT and thus decrease polyamine levels in infected cells. Exhaustion of spermine and spermidine levels due to lowered expression of ODC is also a feature of lytic KSAV [54] and Coxsackie virus B3 (CVB3) [58] infections. So, our data expand the list of viral pathogens that deregulate the expression of polyamine-metabolizing enzymes and decrease polyamine content.

Polyamines are important for the replication of various viruses. In recent years, the Mounce group demonstrated that polyamines ensure the infectivity of virions either by direct incorporation into the virus particle [59] or by incorporation of cholesterol [60], thus conferring binding of virions to the cell surface and concomitant virus entry [59,61,62]. During polyamine exhaustion, non-infectious viral particles can be formed, as shown for Bunyaviruses [63]. So, it is not surprising that polyamine biosynthesis inhibitors such as DFMO or SAM486A exhibit activity towards members of the Filo- [64], Herpes- [54], Entero- [65], Picorna- [65], Alpha- [66], Corona- [61,65], Flavi- [65], Bunya- [65], Rhabdo- [65], and Hepadnaviridae [67] families. However, in all these cases, their mechanism of antiviral action involved the exhaustion of polyamine levels in cells. In this study, to our great surprise, the inhibitory activity of the compounds was accompanied by an increase in the polyamine pool. The mechanism by which polyamines suppress HCV replication is not fully understood. On one hand, spermine and spermidine stimulate RNA polymerase activity of the HCV NS5B protein; however, at the same time, they inhibit RNA unwinding by the viral helicase [68].

Suppression of enteroviruses was earlier also shown for DENSpm [69]. Again, our study shows that these inhibitors also demonstrate anti-HCV activity and add APA to the list of antiviral agents. Moreover, we show that HCV replication can be inhibited by the inhibitor of polyamine oxidases MDL72.527, which was previously studied only for Ebola virus [70].

Polyamines also support the replication of viruses by ensuring the hypusination of eIF5A. KSAV [51,54], Coxsackie virus B3 [71], vesicular stomatitis (VSV), influenza A, Zika, and Chikungunya viruses [72] increase levels of hypusinated eIF5A. This factor enhances the translation of the Ebola genome [73] and HIV transcription [74]. Inhibitors of deoxyhypusine synthase and deoxyhypusine hydrolase also exhibit a wide spectrum of antiviral activity [54,61,64,72,74]. Interestingly, neither of them had an antiviral effect on HCV in our study.

One of the most unexpected results of the study is the nonstandard response of Huh7.5 cells and especially the cells harbouring HCV replicon to the treatment with compounds that target polyamine-metabolizing enzymes such as DENSpm and especially DFMO. First, Huh7.5 cells demonstrated high resistance to DENSpm: this compound caused only a moderate reduction in spermine and spermidine levels upon treatment, and the cells remained viable even upon treatment with 300 µM of the drug (Figure S1). Second, the cells also demonstrated resistance to DFMO, which is an irreversible inhibitor of ODC: at subtoxic concentrations, this drug decreased intracellular ODC activity by two-fold only. Third, the treatment of cells containing the HCV replicon led to an unprecedented elevation of the levels of both spermine and spermidine. It could be speculated that the effect may be due either to enhanced polyamine import from serum in the culture medium or by some nonstandard feedback mechanism by which the cells counteract polyamine depletion. Such an effect was previously described by J. Janne’s group, which raised DFMO-resistant human and murine leukaemia cell lines by maintaining cells in the presence of subtoxic drug concentrations and then observed either multiplication of the ODC1 gene or its enhanced transcription [75]. In the presence of DFMO, this cell line enhanced ODC expression to counteract the inactivation of the enzyme by the drug. Although we have not quantified ODC1 copy numbers in Huh7.5 cells with the HCV replicon, it cannot be excluded that ODC1 multiplication occurs during the selection of the cells, possibly mediated via HCV-enhanced ROS production [31,70].

Here, we show that HCV decreases the expression of ARGI and thus causes accumulation of its substrate arginine in the infected Huh7.5 cells. So far, the data on expression of Arginase 1 in the context of HCV infection have been discrepant. Several groups did not find any changes in Arg1 expression in liver samples of chronic hepatitis C virus patients without signs of steatosis [76,77], while others observed increased staining of the enzyme in biopsy sections [78]. However, Arg1 down-regulation was also reported for Huh7.5 cells harbouring the full-length HCV replicon [79]. Decreased expression of Arg1 and concomitant accumulation of arginine could be the feature of hepatocarcinoma cells during non-alcoholic steatohepatitis and metabolic syndrome [80]. So, we cannot rule out that down-regulation of Arg1 during HCV expression can contribute to viral pathogenesis.

Cell metabolism is affected not only by the virus but also by the culture medium. Almost all studies are performed in cell lines maintained in classical media (such as MEM, DMEM, RPMI, F12, etc.) formulated in the 1950s or early 1960s [46]. Their recipes were to ensure rapid growth of cell biomass and longer period between the replacement of conditioned medium with a fresh one. However, several lines of evidence have appeared during the last decade suggesting that the metabolism of cells maintained in classical medium sometimes does not mimic the processes in vivo (summarised in [46]). One of the most pronounced differences is the dependence of various tumour cell lines on glutaminolysis when grown in DMEM or other “old” medium, with no noticeable effect on the antitumor/antiproliferative activity of a glutaminase 1 inhibitors in vivo or in 3D cell cultures [81,82]. The other example is an inverted urea cycle in cells maintained in DMEM-F12 medium like the one used in our study: arginine is converted not into ornithine or citrulline but into argininosuccinate, albeit with different efficiency in different cell lines [35]. Therefore, several groups recently developed media (HPLM, Plasmax) that resemble human blood plasma and showed “normalization” of the urea cycle [83,84]. So, we used Plasmax medium to verify the changes in cell metabolism in HCV-infected cells. Indeed, the key event, i.e., the suppressed expression of arginase 1, was consistent in the case of both media.

Proline biosynthesis and catabolism may be not only the independent processes that regulate steady-state proline concentrations but also form a proline cycle [85]. This cycle is formed by the conversion of the amino acid into Δ^1^-pyrrolidine-5-caboxylate (P5C) in mitochondria by PRODH, the export of this metabolite into the cytoplasm, and the subsequent reverse transformation into Pro by Δ^1^-pyrrolidine-5-carboxylate reductase 3 (P5CR3, P5CRL). This cycle both feeds oxidative phosphorylation in mitochondria by providing electrons from the PRODH-reduced flavin (FADH2) and supports nucleotide biosynthesis via oxidation of NADPH into NADP^+^ by P5CRL. So, enhanced metabolic flux of proline and P5C may be a mechanism of activation of other metabolic pathways. However, up-regulation of PRODH in the HCV-infected cells is not accompanied by changes in the expression of proline biosynthetic enzymes, and of P5CRL in particular, thus excluding the importance of the proline cycle in the regulation of the pentose phosphate pathway during HCV infection. Of note, PRODH expression is positively regulated by AMPK, p53, and PPARγ, and negatively by cMyc transcription factors. Since HCV is known to activate cMyc [20,86] and suppress p53 [87] and AMPK signalling [83], they are unlikely to mediate up-regulation of PRODH during the infection. This points to PPARγ as the likely regulator of PRODH expression in HCV-infected cells.

Polyamines are synthesized from a non-proteinogenic amino acid, ornithine, which in turn can be produced either from arginine in the urea cycle or from glutamic semialdehyde (GSA) by ornithine aminotransferase (OAT). The latter is formed either via a non-enzymatic isomerization of P5C (i.e., from proline catabolism) or from glutamate by Δ^1^-pyrrolidine-5-carboxylate synthase (encoded by the ALDH18A1 gene) (i.e., from glutaminolysis). A few papers have suggested that neoplastic transformation may switch the origin of ornithine from arginine to GSA, although these data were obtained from non-liver cells [88]. Since HCV activates both glutaminolysis [20] and proline catabolism and suppresses the conversion of arginine into ornithine (this study), it is tempting to speculate that this virus also reprograms the routes of ornithine production. Although the origins of ornithine were not accessed using flux analysis by mass spectrometry using ^13^C-labelled ornithine and glutamate/proline, we used an inhibitor of OAT—L-canaline. However, this compound only slightly affected the viability of HCV-infected cells (Figure S1) and did not affect HCV replication levels, thus not supporting our assumption.

Arginine–glycine amidinotransferase (GATM, AGAT) is an enzyme that catalyses an alternative arginine-to-ornithine conversion pathway. Its second product, guanidinoacetate, is a precursor for the biosynthesis of creatine and creatinine by GAMT. It is generally accepted that the first reaction takes place in the kidney and the second in the liver. However, GATM mRNA levels in the liver in hepatocarcinoma Huh7.5 cells, as well as in nontumor hepatocyte-like HepaRG cells, are at least six-fold higher than those of arginase 1 [89]. So, we cannot exclude that in HCV-infected cells ornithine is synthesized via a GATM-mediated reaction.

Agmatinase is the least studied enzyme of polyamine biosynthesis in mammalian cells. In prokaryotic, plant, and fungi cells, agmatinase produces ornithine from agmatine generated from arginine by arginine decarboxylase (ADC). Since mammals were thought not to encode ADC in their genome, almost no attempts to identify arginase in mammalian cells were made. In 2002, this gene was identified in HepG2 cells, and up-regulation of its transcription in the context of HBV infection was reported [90]. Later, ADC was also discovered in mammalian brain cells [91]. However, the biological significance of agmatinase in mammals remains obscure since its substrate agmatine can originate either from gut biota or from food, as ADC has very low expression levels in a majority of tissues. Nevertheless, we included this gene in our analysis and demonstrated the absence of its regulation in HCV-infected cells.

## 5. Conclusions

To sum up, we have demonstrated that HCV perturbs several metabolic pathways, including the urea cycle, and polyamine and proline metabolism. Biosynthesis and degradation of spermine and spermidine are critical for the virus, as pharmacological inhibitors of the respective enzymes display pronounced antiviral activity. Replenishment of proline levels also suppressed HCV replication.

This study has several limitations. First, our data have been obtained only from in vitro models (HCVcc and subgenomic replicons). These models are based on a unique virus isolate from a patient with fulminant hepatitis, which is the predominant strain that supports the efficient production of infectious particles in cells and does not require adaptive mutations for replication in cell culture [92,93]. These systems are also based on a unique hepatoma cell line, Huh7.5, with a defect in innate immune responses, but again, it is the only cell line that ensures efficient HCV replication [94]. Thus, these data need to be reproduced in vivo. In addition, further studies are required to unveil how these changes impact the development of the pathology associated with chronic HCV infection.

## Figures and Tables

**Table 1:** Influence of the compounds on polyamine metabolism.

Cell Line	Compound	Polyamine (nmol/mg DNA)		Enzyme Activity (nmol/h/mg Total Protein)	
		Spermidine	Spermine	ODC	SSAT
Naïve Huh7.5	Control	55.3 ± 10.8	49.6 ± 8.3	9.3 ± 1.2	33.1 ± 2.6
	DENSpm	41.8 ± 9.5	17.9 ± 3.9	54.7 ± 3.2	217.5 ± 18.6
	DFMO	47.0 ± 10.1	37.2 ± 5.4	5.5 ± 0.6	2.7 ± 1.5
	MDL72.527	63.5 ± 13.8	51.2 ± 13.7	4.4 ± 0.7	2.3 ± 0.2
HCV replicon-harbouring Huh7.5 cells	Control	38.7 ± 6.1	27.9 ± 10.6	4.3 ± 1.1	8.6 ± 0.8
	DENSpm	33.3 ± 8.3	14.1 ± 7.4	38.7 ± 2.1	190.3 ± 10.7
	DFMO	177.3 ± 42.2	140.1 ± 52.1	7.1 ± 1.5	1.2 ± 0.1
	MDL72.527	227.6 ± 39.4	177.6 ± 27.8	17.4 ± 3.3	1.2 ± 0.2

## Data Availability

The raw data supporting the conclusions of this article will be made available by the authors upon request.

## Supplementary Materials

The following supporting information can be downloaded at https://www.mdpi.com/article/10.3390/cells13121036/s1, Table S1: Oligonucleotides used in the study; Figure S1: Inhibitors of ornithine decarboxylase.
