# Supplementary material for: Hepatitis C Virus Dysregulates Polyamine and Proline Metabolism and Perturbs the Urea Cycle
Source: Cells. 2024 Jun 14;13(12):1036. doi: 10.3390/cells13121036 (PMC11201506; doi:10.3390/cells13121036)
Supplement: Supplementary file 1 [file cells-13-01036-s001.zip › cells-3011553-supplementary.pdf]

Supplementary materials for

Hepatitis C virus dysregulates polyamine and proline metabolism and perturbs urea cycle

Natalia F. Zakirova [1,†], Olga A. Khomich [1,2,†], Olga A. Smirnova [1,†], Jennifer Molle [2], Sarah Duponchel [2], Dmitry V. Yanvarev [1],Vladimir T. Valuev-Elliston [1], Lea Monnier [2], Boyan Grigorov [2], Olga N. Ivanova [1], Inna L. Karpenko [1], Mikhail V. Golikov [1], Cedric Bovet [3], Barbara Rindlisbacher [3], Alex R. Khomutov [1], Sergey N. Kochetkov [1], Birke Bartosch [2] and Alexander V. Ivanov [1,*]

[1] Engelhardt institute of molecular biology, Russian Academy of Sciences, Moscow, Russia; nat_zakirova@mail.ru (N.Z), oakhomich@gmail.com (O.K.), osmirnovaimb@gmail.con (O.S.); yanvarev@eimb.ru (D.Y.), gansfaust@mail.ru (V.V.-E.), olgaum@yandex.ru (O.I.), ilkzkil@gmail.com (I.K.), cool.mik3492594@yandex.ru (M.G.), alexkhom@list.ru (A.K.), snk1952@gmail.com (S.K.), aivanov@yandex.ru (A.I.)

[2] Université Claude Bernard Lyon 1, INSERM 1052, CNRS 5286, Centre Léon Bérard, Centre de recherche en cancérologie de Lyon, Lyon, 69434, France; jennifer.molle@inserm.fr (J.M), lea_monnier@yahoo.com (L.M.), boyan.grigorov@inserm.fr (B.G.), birke.bartosch@inserm.fr (B.B.)

[3] University Institute of Clinical Chemistry, Inselspital, Bern University Hospital, University of Bern, Switzerland; cedric.bovet@protonmail.com (C.B.), barbara.rindlisbacher@medics.ch (B.R.)

* Correspondence: aivanov@yandex.ru; Tel.: +7-499-135-6065

† These authors contributed equally to this work.

Table S1. Oligonucleotides used in the study

Gene ID	Orientation	Sequence[d]	Product length
HCV	Forward	5'-GTCTAGCCATGGCGTTAGTA-3'	246
	Reverse	5'-CTCCCGGGGCACTCGCAAGC-3'	
ODC	Forward	5′-TTGCGGATTGCCACTGATGATTCC-3′	186
	Reverse	5′-ATCAGAGATTGCCTGCACGAAGGT-3′	
SSAT	Forward	5′-ATCTAAGCCAGGTTGCAATGA-3′	189
	Reverse	5′-GCACTCCTCACTCCTCTGTTG-3′	
SMOX	Forward	5′-GATCCCGGCGGACCATGTGATTGTG-3′	576
	Reverse	5′-CCTGCATGGGCGCTGTCTTTG-3′	
PAOX	Forward	5′-GTCACCGTGCCCTTAGGTT-3′	103
	Reverse	5′-TCCCAAAGCCTATCTTCCTG-3′	
AMD	Forward	5′-AAGGATCTGGGGATCTTCGCA-3′	100
	Reverse	5′-TGCTTGTCAGTTTTTGTCACAC-3′	
OAZ1	Forward	5′-AGAGGAACTAACGTCCAACGAC-3′	225
	Reverse	5′-TTCTTGTGGAAGCAAATGAAGA-3′	
ArgI	Forward	5′-TGATGTGAAGGATTATGGGGAC-3′	272
	Reverse	5′-TGGTTGTCAGTGGAGTGTTG-3′	
ArgII	Forward	5′-GTATCCAGAAGGTCATGGAACG-3′	199
	Reverse	5′-AGTGCTGATAGCAACCCTGT-3′	
OTC	Forward	5′-TGCAAGGGAAGTCCTTAGGC-3′	89
	Reverse	5′-TCCTCCCAGAAGTGCAAAGC-3′	
ASS	Forward	5′-CAACACCCCTGACATTCTCG-3′	396

	Reverse	5′-ACTTTCCCTTCCACTCGCTC-3′	
ASL	Forward<br>Reverse	5′-TCCCAGCACCTACAACAAAGA-3′<br>5′-TAGTCCCACACGCAGATCACG-3′	339
CPS	Forward<br>Reverse	5′-CTGACCCTGCCTACAAAG-3′<br>5′-CACCAGCAAACCTGAAAC-3′	146
OAT	Forward<br>Reverse	5′-CTGCCGTAAGAGGAAAAGGA-3′<br>5′-GCTTGGCCAGAAGTCCATTA-3′	111
PRODH	Forward<br>Reverse	5′-CAACGCCATGTACCACAGGT-3′<br>5′-ATGCCTAGCAGCTGTCCAAA-3′	180
PYCR1	Forward<br>Reverse	5′-ACACCCCACAACAAGGAGAC-3′<br>5′-CTGGAGTGTTGGTCATGCAG-3′	220
P5CS1<br>(ALDH18A1)	Forward<br>Reverse	5′-TCTTCTGCCCTGGGTCAAGT-3′<br>5′-AAACGGGATGTTGCTCCAAGA-3′	97
P5CDH<br>(ALDH4A1)	Forward<br>Reverse	5′-CCATCTCGCCCTTTAACTTCAC-3′<br>5′-ACTGGGCTTCCATAGGACCA-3′	89
AZIN1	Forward<br>Reverse	5′-ATTGATGATGCAAACTACTCCGT-3′<br>5′-GCCACTACATTCTGCCATTGA-3′	167
GUS	Forward<br>Reverse	5'-CGTGGTTGGAGAGCTCATTTGGAA-3'<br>5'-ATTCCCCAGCACTCTCGTCGGT-3'	73

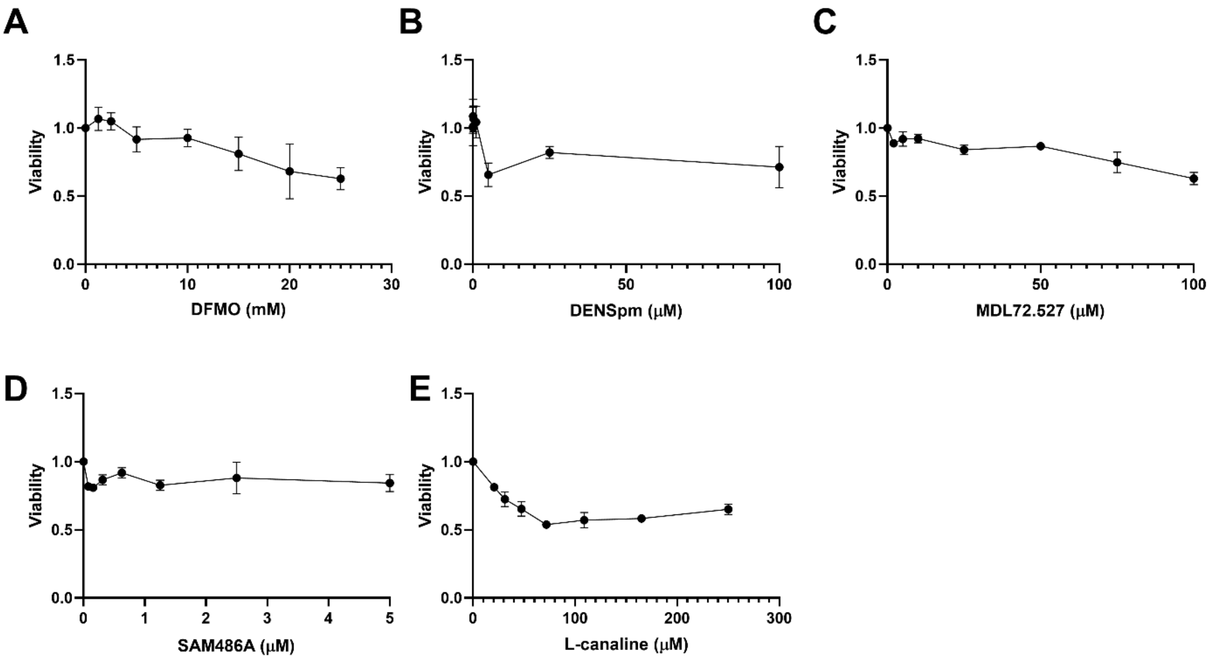

Figure S1. Inhibitors of ornithine decarboxylase
